# Demonstrating trustworthiness when collecting and sharing genomic data: public views across 22 countries

**DOI:** 10.1186/s13073-021-00903-0

**Published:** 2021-05-25

**Authors:** Richard Milne, Katherine I. Morley, Mohamed A. Almarri, Shamim Anwer, Jerome Atutornu, Elena E. Baranova, Paul Bevan, Maria Cerezo, Yali Cong, Alessia Costa, Christine Critchley, Josepine Fernow, Peter Goodhand, Qurratulain Hasan, Aiko Hibino, Gry Houeland, Heidi C. Howard, S. Zakir Hussain, Charlotta Ingvoldstad Malmgren, Vera L. Izhevskaya, Aleksandra Jędrzejak, Cao Jinhong, Megumi Kimura, Erika Kleiderman, Brandi Leach, Keying Liu, Deborah Mascalzoni, Álvaro Mendes, Jusaku Minari, Dianne Nicol, Emilia Niemiec, Christine Patch, Jack Pollard, Barbara Prainsack, Marie Rivière, Lauren Robarts, Jonathan Roberts, Virginia Romano, Haytham A. Sheerah, James Smith, Alexandra Soulier, Claire Steed, Vigdis Stefànsdóttir, Cornelia Tandre, Adrian Thorogood, Torsten H. Voigt, Nan Wang, Anne V. West, Go Yoshizawa, Anna Middleton

**Affiliations:** 1 Society and Ethics Research Group, Wellcome Connecting Science, Wellcome Genome Campus, Cambridge, CB10 1SA UK; 2 Department of Public Health and Primary Care, University of Cambridge, Cambridge, CB2 0SR UK; 3 RAND Europe, Cambridge, CB4 1YG UK; 4 Institute of Psychiatry, Psychology & Neuroscience, King’s College London, London, SE5 8AF UK; 5 Centre for Epidemiology and Biostatistics, Melbourne School of Global and Population Health, The University of Melbourne, Melbourne, 3010 Australia; 6 Wellcome Sanger Institute, Cambridge, CB10 1SA UK; 7 Department of Forensic Science and Criminology, Dubai Police GHQ, Dubai, United Arab Emirates; 8 Keynote IAS, New Delhi, 110060 India; 9 Russian Medical Academy of Continuous Professional Education, Moscow, 119049 Russia; 10 EMBL-EBI, Wellcome Genome Campus, Cambridge, CB10 1SA UK; 11 Medical Ethics Program, Peking University Health Science Center, Beijing, 100191 China; 12 Department of Psychological Sciences, Swinburne University of Technology, Melbourne, 3122 Australia; 13 Centre for Law and Genetics, University of Tasmania, Hobart, 7001 Australia; 14 Centre for Research Ethics & Bioethics (CRB), Uppsala University, SE-751 22 Uppsala, Sweden; 15 Ontario Institute for Cancer Research, MaRS Centre, Toronto, M5G 0A3 Canada; 16 Department of Genetics & Molecular Medicine, Kamineni Hospitals, Hyderabad, 500 068 India; 17 SAAZ Genetics, Hyderabad, 500033 India; 18 Faculty of Humanities and Social Sciences, Hirosaki University, Hirosaki, 036-8560 Japan; 19 Medical Ethics, Lund Universitet, Sölvegatan, 19 Lund, Sweden; 20 Department of Public Health and Caring Scienec, Uppsala University, 751 22 Uppsala, Sweden; 21 Department of Molecular Medicine and Surgery, Karolinska Institutet, 171 76 Solna, Sweden; 22 Research Centre for Medical Genetics, Moscow, 115522 Russia; 23 Independent Scholar, Warsaw, Poland; 24 Department of Epidemiology and Biostatistics, School of Health Sciences, Wuhan University, Wuhan, 430071 China; 25 Institute of Innovation Research, Hitotsubashi University, Tokyo, 186-8603 Japan; 26 Centre of Genomics and Policy, McGill University, Montreal, H3A 0G1 Canada; 27 Public Health, Department of Social Medicine, Osaka University Graduate School of Medicine, Osaka, 565-0871 Japan; 28 School of Public Health, Peking University Health Science Center, Beijing, 100191 China; 29 EURAC, Institute of Biomedicine, 39100 Bolzano, Italy; 30 UnIGENe and CGPP – Centre for Predictive and Preventive Genetics, IBMC – Institute for Molecular and Cell Biology, i3S – Instituto de Investigação e Inovação em Saúde, Universidade do Porto, 4200-135 Porto, Portugal; 31 Uehiro Research Division for iPS Cell Ethics, Center for iPS Cell Research and Application (CiRA), Kyoto University, Kyoto, 606-8507 Japan; 32 Genomics England, Queen Mary University of London, London, EC1M 6BQ UK; 33 Department of Political Science, University of Vienna, 1010 Vienna, Austria; 34 Department of Global Health & Social Medicine, King’s College London, London, WC2R 2LS UK; 35 DILTEC, Sorbonne Nouvelle, 75005 Paris, France; 36 Landspitali, the National University Hospital of Iceland, 101 Reykjavík, Iceland; 37 Institute of Sociology, RWTH Aachen University, 52062 Aachen, Germany; 38 Indiana University Maurer School of Law, Bloomington, 47405 USA; 39 Work Research Institute (AFI), Oslo Metropolitan University, 0130 Oslo, Norway; 40 Faculty of Education, University of Cambridge, Cambridge, CB2 8PQ UK

## Abstract

**Background:**

Public trust is central to the collection of genomic and health data and the sustainability of genomic research. To merit trust, those involved in collecting and sharing data need to demonstrate they are trustworthy. However, it is unclear what measures are most likely to demonstrate this.

**Methods:**

We analyse the ‘Your DNA, Your Say’ online survey of public perspectives on genomic data sharing including responses from 36,268 individuals across 22 low-, middle- and high-income countries, gathered in 15 languages. We examine how participants perceived the relative value of measures to demonstrate the trustworthiness of those using donated DNA and/or medical information. We examine between-country variation and present a consolidated ranking of measures.

**Results:**

Providing transparent information about who will benefit from data access was the most important measure to increase trust, endorsed by more than 50% of participants across 20 of 22 countries. It was followed by the option to withdraw data and transparency about who is using data and why. Variation was found for the importance of measures, notably information about sanctions for misuse of data—endorsed by 5% in India but almost 60% in Japan. A clustering analysis suggests alignment between some countries in the assessment of specific measures, such as the UK and Canada, Spain and Mexico and Portugal and Brazil. China and Russia are less closely aligned with other countries in terms of the value of the measures presented.

**Conclusions:**

Our findings highlight the importance of transparency about data use and about the goals and potential benefits associated with data sharing, including to whom such benefits accrue. They show that members of the public value knowing what benefits accrue from the use of data. The study highlights the importance of locally sensitive measures to increase trust as genomic data sharing continues globally.

**Supplementary Information:**

The online version contains supplementary material available at 10.1186/s13073-021-00903-0.

## Background

The future of genomic medicine relies on the ability of researchers and clinicians to access large quantities of genomic and health data. The support of patients and the public for the collection and use of data is central to the success and sustainability of genomic research [1]. However, public willingness to share data and trust in the bodies responsible for the collection and sharing of genomic data varies between countries and between actors involved in the genomic data ecosystem [2, 3]. Trust in the for-profit research sector and governments, for example, is commonly lower than that in non-profit and clinical organisations [4–7]. In this paper, we present findings on public views of measures that may increase trust by ensuring or demonstrating the *trustworthiness* of the organisations, institutions and individuals working with genomic datasets.

The shift in focus from trust to trustworthiness is an important one, recognised in a growing body of work on genomic medicine [2, 8]. Trust involves a relationship between two actors with an expectation of an outcome [9]. Discussion of trust often places the emphasis on the one placing trust, whether a patient or a member of the public. However, these individuals are placing trust that another actor—for example a clinician, researcher or company—is motivated to act to pursue a particular goal [10]. It is here that trustworthiness is critical. If these actors are not trustworthy, trust is neither merited nor meaningful. Trust misplaced in this way has the potential to harm both those who place their trust and those who betray it, for example through long-term impact on reputation or the loss of future research opportunities.

An emphasis on trustworthiness moves the focus away from the public to those involved in collecting and using data and presents an opportunity for the latter to act to exhibit qualities that demonstrate that they are worthy of trust [11]. The meaning of trustworthiness in practice, however, remains unclear, including the activities or measures that show that those collecting and using data are worthy of trust [2, 12–14]. A number of features exhibited by ‘trustworthy’ systems for genomic data have been suggested. These include the importance of establishing shared values and common goals and motives between researchers and participants or members of the public involved in research. This may include demonstrating a focus on the common good and the equitable distribution of risks and benefits [15]. It may also mean supporting research ethics measures such as informed consent with robust governance that is responsive to stakeholders, respectful, transparent, sustainable, audited and regularly assessed, potentially combined with legal protections [1, 16–19]. Further, work to embed research in relation to local values and goals may be particularly important in addressing potential distrust arising from historical discrimination in healthcare and research [10, 16].

This prior work identifies practices that are already in place in at least some large genomic data initiatives and those to which they and other initiatives might aspire—some that might be new, others that may involve refinement of existing activities. To date, however, discussions of the trustworthiness of genomic and health data initiatives and their relationship with public trust have rarely enabled comparisons between contexts, particularly between countries [3, 5, 20]. This makes it difficult to consider how transferable measures to establish trustworthiness might be and how they are differently valued in different research contexts. Such an international perspective is critical given the need for international data sharing and has the potential to support the development of policies for the sharing of genomic data through initiatives like the Global Alliance for Genomics and Health (GA4GH) [21].

We provide an international perspective through an analysis of the ‘Your DNA, Your Say’ online survey, a study of public perspectives on genomic data sharing that draws on responses from 36,268 individuals across 22 low-, middle- and high-income countries, gathered in 15 languages. We have previously reported on variations in trust within and between these countries, and the relationship between trust and willingness to donate DNA and health information [6, 22]. Here we focus in detail on responses to a question asking participants, who would consider donating DNA or medical information, which measures would help them trust those with whom their data may be shared. Our aim with this question is to draw out how current and potential practices contribute to demonstrating the trustworthiness of data users. We analyse how participants perceived the relative value of these measures, how this varied across the 22 countries of the study, and provide a consolidated ranking of the measures. We then examine similarities and differences between countries, clustering countries that share similar perspectives on the value of these measures. This allows us to consider the implications of our findings for data sharing policies and their applicability across social, cultural and regulatory contexts.

## Methods

### Sample

Via the international network of researchers affiliated with the GA4GH, the research team invited social science, genetic counselling, bioethics and policy collaborators around the world to participate in conducting the ‘Your DNA, Your Say’ project, either supporting recruitment into the project and/or translating the survey. For all countries except Japan, Pakistan and India (see below), data were collected using a cross-sectional online survey with participants recruited via market research company Dynata. We aimed to recruit a sample that was as representative as possible of each country’s population with regard to gender, age and education level. To this end, participant characteristics were monitored during recruitment to proactively include individuals from under-represented population subgroups. Sociodemographic characteristics of participants from each country are shown in Additional file 1: Table S1 and Additional file 1: Figures S1-S5.

In Japan, participants were recruited through a survey research company (Cross Marketing) using the same approach. In Pakistan and India, recruitment was conducted by market research companies (Foresight and Maction, respectively), and methods were varied to account for lower Internet access. In Pakistan, participants completed the questionnaire on a tablet at a central location rather than at home. In India, participants completed the questionnaire on tablets provided by field researchers. Completed surveys were gathered from Argentina, Australia, Belgium, Brazil, Canada, China, Egypt, France, Germany, India, Italy, Japan, Mexico, Pakistan, Poland, Portugal, Russia, Spain, Sweden, Switzerland, UK and the USA. Participants were paid a small financial reward (<£1) for participating, and due to the nature of the recruitment, there are no details on the non-response rate. The study methodology, design, recruitment strategy, limitations and process of data collection are described separately [23].

### Measures

**Fig. 1:**
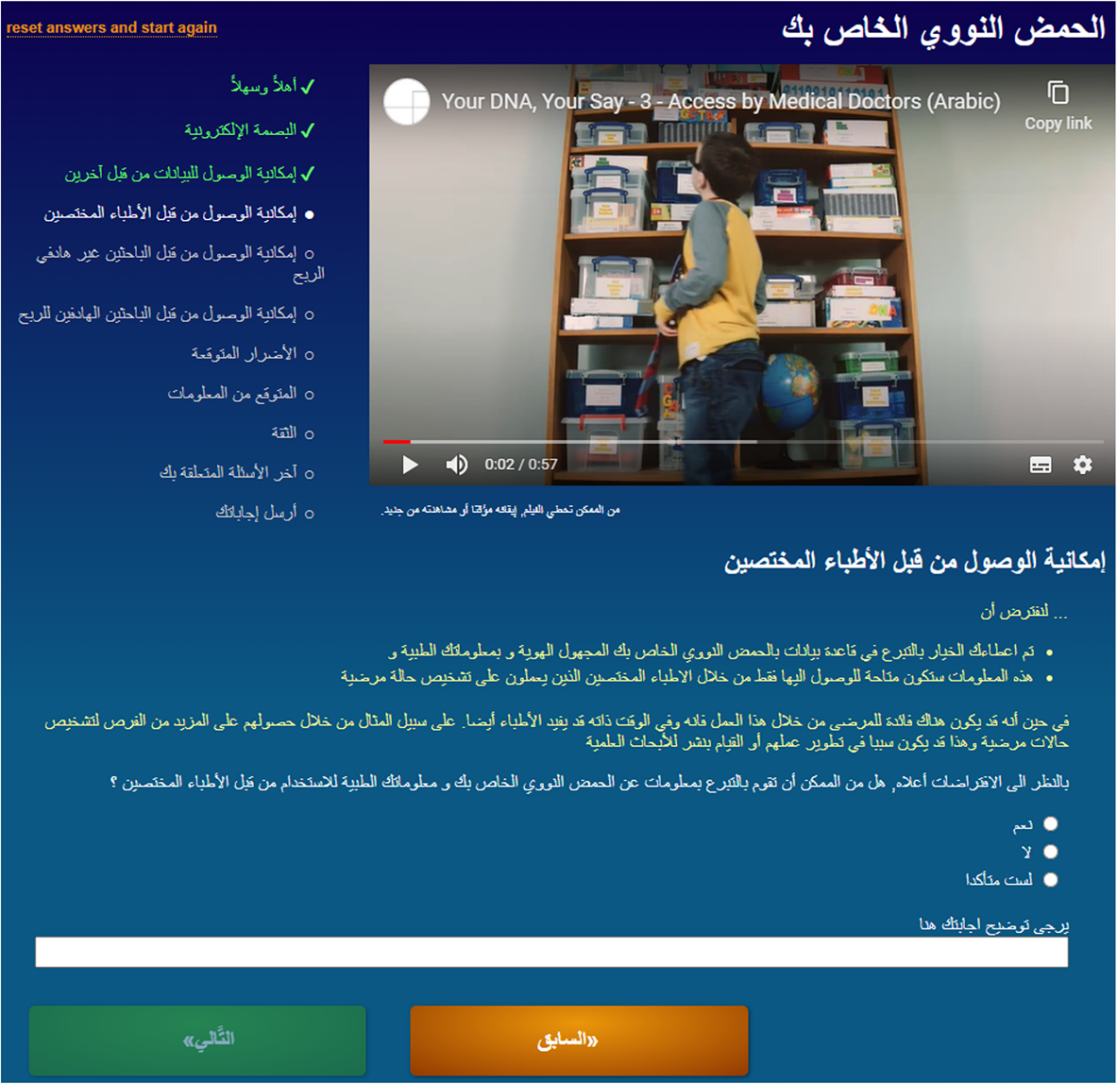
The appearance of the online Your DNA, Your Say survey (Arabic version). The Your DNA, Your Say questionnaire was presented in 15 languages. Background information on genomic research and data sharing was provided by nine films

Our online survey can be accessed from www.YourDNAYourSay.org. It contains 29 questions; background information about the landscape of genomic research and data sharing is provided via nine films that sit within the survey (see Fig. 1); no prior knowledge about genomics is required to participate.

In this paper, we analyse a question that asked participants what information would help them to trust the people asking them to donate DNA information and/or medical information. As shown in Table 1, this question allowed for a structured response, with participants selecting from a range of measures suggested in existing work on the ethics and governance of genomic data platforms. These measures include practices already in place in some, if not many, genomic data initiatives, and those that are more aspirational [24].

**Table 1:** YDYS survey question relating to measures that would help people to trust recipients of donated DNA/medical information

Q: What information would help you to trust the people asking you to donate DNA information and/or medical information? (choose all that apply)	• Transparent information about WHO will benefit from the data access • Transparent information about HOW others will benefit personally, professionally and commercially from the data access • A website that clearly explains the pros and cons of data access • The option to opt out of having your information accessed by other researchers • The option to withdraw your information in the future • Biographies and photos of the sorts of researchers who would access the data • Knowing exactly who is using your information, and for what purpose • The ability to access your own DNA information and/or medical information • Being able to communicate directly with gatekeepers of my DNA information and/or medical information • Details about the sanctions applicable if my data is misused by others • Other, please provide: • I would not donate my DNA information and/or medical information

One feature of relevance to this question is how we presented information about motives associated with data donation and collection; in the survey films and text, we articulated how different actors may obtain and be motivated by disparate multiple and divergent benefits from the use of donated data. When asking whether participants would be willing to donate their DNA and medical information to medical doctors for use in making a diagnosis in another patient, they were told: ‘Whilst there might be benefits to patients from this work, medical doctors might benefit too. For example, through getting more diagnoses for patients and therefore being better at their jobs or getting scientific publications’. When asking whether participants would be willing to donate their DNA and medical information for use by non-profit researchers doing research, for example on how DNA links to disease, they were told: ‘There might be benefits to society from this work. But also, individual researchers and organisations might benefit too. For example, individual researchers could advance their career and organisations bring in new funding’. Then finally, when asking whether participants would be willing to donate their DNA and medical information for use by for-profit researchers doing research, for example, developing new medicines, they were told: ‘There might be benefits to society from this work. But also, individual researchers and organisations might benefit too. For example, individual researchers might advance their career and companies make a profit’. Thus, we were explicit in giving examples of *how* access and use of data might be motivated by, and lead to, the accrual of personal and organisational, as well as societal and clinical benefit.

We treat countries as the unit of analysis rather than individuals, deriving country-level variables from data collected at the individual level. We use participant-provided information on country of residence and on which measures would help support trust. We focus on those who said that they would potentially be willing to donate DNA or medical information (*n*=29,814); participants who were not willing to donate were excluded from further analyses. The breakdown of refusals by country is shown in Additional file 1: Table S2 (range 4% (India) to 33% (Japan); mean 16.6%).

### Analysis

#### Country-level responses

We converted individual responses to this question into a country-level ranked list of these measures from most to least important. This ranking is the variable of interest. We also calculated a ‘global’ ranked list based on overall responses. This was compared with a consensus-generated list derived as part of the analysis (described below).

#### Consolidated ranking across countries

We used the top-*k* approach [25] to identify common top-ranking responses across countries and to generate a consolidated consensus rank for the measures. We calculated the consensus ranking for the measures using three algorithms (Borda, Markov Chain – majority rule, and the Order Explicit Algorithm) using the *TopKLists* package [26].

#### Correlation of responses between countries

The rank of the different measures was calculated for each country. We examined pair-wise correlations between all countries, estimated using Kendall’s tau-b coefficient, which permits estimation of correlations between non-parametric data with ties. In total, there are 231 unique pair-wise correlations; we used a Bonferroni correction to guide interpretation of significance of correlations. We used the *Superheat* package to visualise the pair-wise correlations and generate a cluster dendrogram illustrating similarities between countries [27].

## Results

**Fig. 2:**
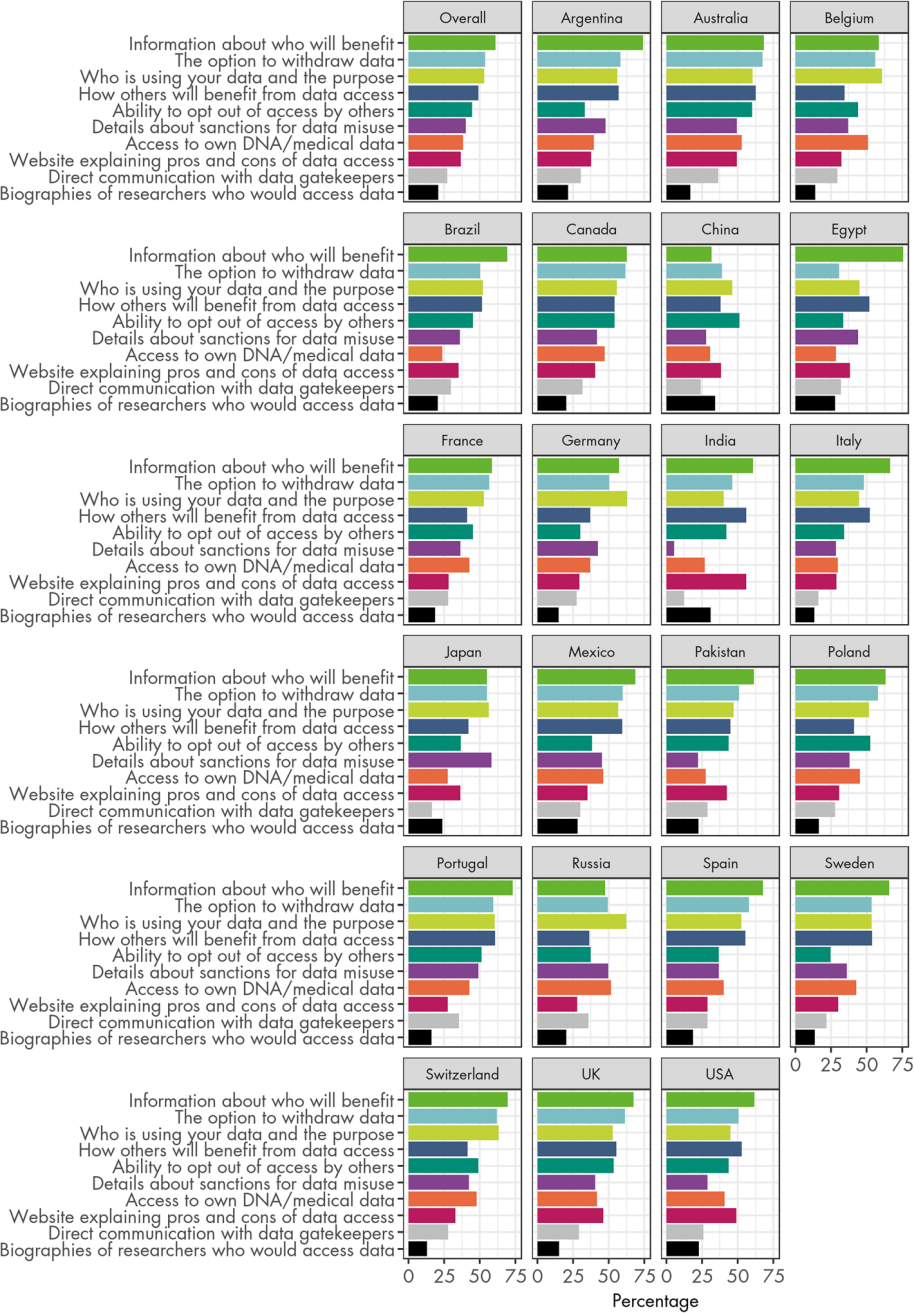
Measures to help trust recipients of donated DNA/medical information. Percentage of participants endorsing each measure proposed to help to trust recipients of donated DNA/medical information, overall and by country

The percentage of participants endorsing each measure to increase trust in the recipients of donated data is shown for the overall sample and by country in Table 2 and Fig. 2. Measures are ordered by ranking in the overall sample.

**Table 2:** Percentage of participants endorsing each measure proposed to help to trust recipients of donated DNA/medical information, overall and by country

Measure	Overall	Argentina	Australia	Belgium	Brazil	Canada	China	Egypt	France	Germany	India	Italy	Japan	Mexico	Pakistan	Poland	Portugal	Russia	Spain	Sweden	Switzerland	UK	USA
Transparent information about WHO will benefit from the data access	61	74	68	58	69	63	32	75	59	57	61	66	55	68	61	63	73	47	68	66	70	67	62
The option to withdraw your information in the future	54	58	67	56	50	62	39	31	57	50	46	48	55	60	51	58	59	49	58	53	62	61	51
Knowing exactly who is using your information, and for what purpose	53	56	61	61	52	56	46	45	53	63	40	45	56	57	47	52	60	62	52	53	63	53	45
Transparent information about HOW others will benefit personally, professionally and commercially from the data access	49	57	62	35	51	54	38	52	41	37	56	52	42	59	45	41	61	36	55	54	42	55	53
The option to opt out of having your information accessed by other researchers	45	33	60	44	45	54	51	34	45	30	42	34	37	38	44	52	51	37	37	25	49	53	44
Details about the sanctions applicable if my data is misused by others	40	48	49	37	36	42	28	44	36	43	5	29	58	45	22	38	49	50	37	36	42	41	29
The ability to access your own DNA and/or medical information	38	40	53	51	24	47	31	28	43	37	27	30	28	46	27	45	43	52	40	43	48	42	41
A website that clearly explains the pros and cons of data access	37	38	49	32	35	40	38	38	28	29	56	29	36	35	42	31	27	28	29	30	33	46	49
Being able to communicate directly with gatekeepers of my DNA and/or medical information	27	30	37	29	30	32	24	32	28	28	13	16	17	30	29	28	35	36	29	22	28	29	26
Biographies and photos of the sorts of researchers who would access the data	21	21	17	14	21	20	34	28	19	15	31	13	24	28	23	17	16	20	19	14	13	15	23
Other	1	1	0	0	1	1	0	2	1	0	1	1	2	1	3	0	0	1	0	1	0	1	1

**Fig. 3:**
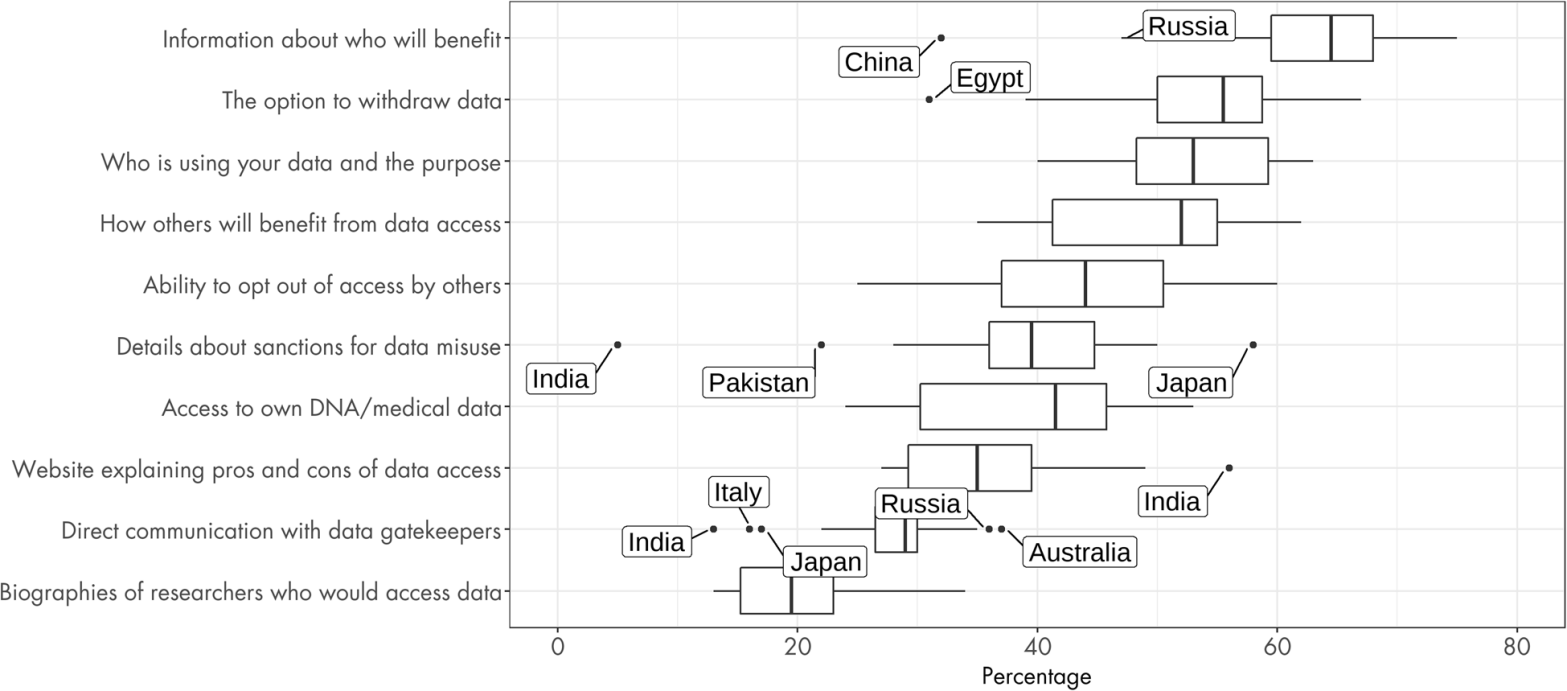
Percentage of participants endorsing each measure by country. Boxplots show the percentage endorsing each measure per country, showing the variability associated with each option. Outliers are labelled

The figure and table illustrate that there is substantial variability between countries in terms of the ranking of the different measures. This is shown further in the boxplots in Fig. 3.

For all but two countries, provision of transparent information about who will benefit from data access was endorsed by a majority (i.e. > 50%) of participants, including more than 70% of respondents in Egypt, Argentina, Portugal and Switzerland, although China was an extreme outlier with only 32% of respondents endorsing this. Overall, the option to withdraw information in the future was the second most endorsed measure across the sample, with the greatest number of respondents endorsing this option in Australia, Canada, Switzerland and the UK, but only 31% in Egypt.

Endorsement was most variable for details about sanctions for misuse of data, ranging from 5% in India to almost 60% in Japan, where it was the most chosen option. Direct interaction with gatekeepers was a divisive measure with many outliers both above (Australia, Russia, Portugal) and below (Italy, Japan, India) the average endorsement.

Countries varied in the number of measures that were endorsed by a majority of respondents. While five or more measures were endorsed by more than 50% of respondents in the UK, Portugal, Canada and Australia, only one—the ability to opt out of having information access by other researchers—was endorsed by an equivalent proportion of respondents in China.

### Consolidated ranking

The consensus results from the top-*k* approach (Table 3) confirm the rank order obtained from the overall ranking based on percentage endorsement (from most to least important). The only exception is that the ability to access one’s own DNA/or medical information is ranked one place above details about sanctions (as opposed to one rank lower based on percentage endorsement).

**Table 3:** Consolidated ranking of measures to increase trust based on the top-*k* approach

1. Transparent information about WHO will benefit from the data access 2. The option to withdraw your information in the future 3. Knowing exactly who is using your information, and for what purpose 4. Transparent information about HOW others will benefit personally, professionally and commercially from the data access 5. The option to opt out of having your information accessed by other researchers 6. The ability to access your own DNA and/or medical information 7. Details about the sanctions applicable if my data is misused by others 8. A website that clearly explains the pros and cons of data access 9. Being able to communicate directly with gatekeepers of my DNA and/or medical information 10. Biographies and photos of the sorts of researchers who would access the data	

### Correlation of responses between countries

**Fig. 4:**
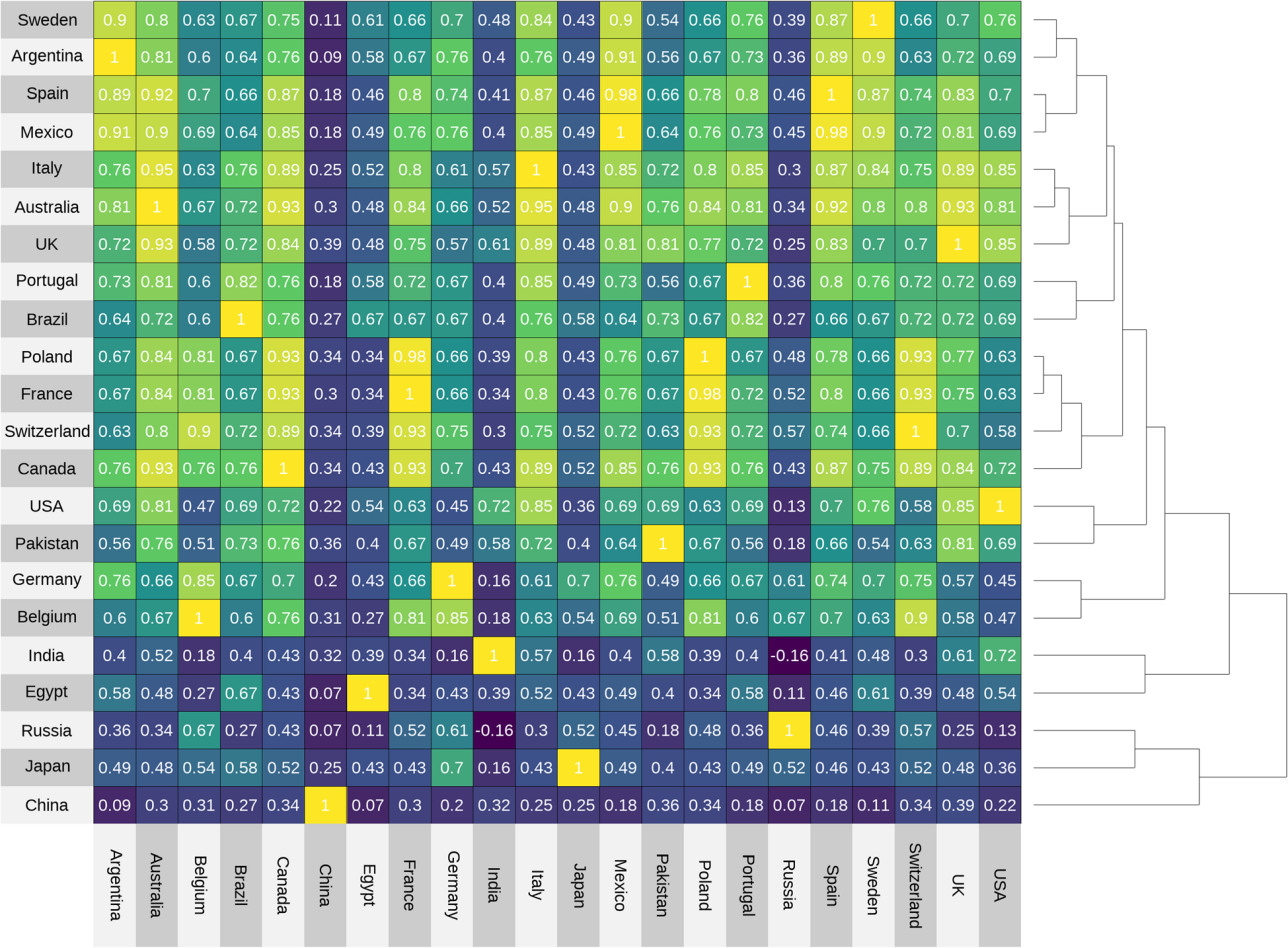
Heatmap of pair-wise correlation estimates. The strength of the pairwise correlation is indicated by the colour of the square, from dark blue to yellow. The dendrogram indicates clustering among countries where responses were closely aligned

The pair-wise correlation estimates for all country pairs are shown in Fig. 4. Pairwise correlations are shown in Table S3 for all estimates greater than or equal to 0.9. The two strongest correlations, with *p* values below the Bonferroni-corrected threshold of 0.0002, were between Mexico and Spain, and France and Poland (both at 0.98). The lowest correlation (data not shown) was between Russia and India, with an estimate of − 0.16.

Overall, the heatmap and dendrogram in Fig. 4 show groups of countries for which the rankings of these measures were very similar and that form specific clades. Similarity is not necessarily related to geographical proximity, as for example in the similarity of Spain and Mexico or the UK and Australia. Some countries, notably China and Russia and to some extent India, Egypt, and Japan have consistently lower correlations with most other countries and are linked only at higher levels of the dendrogram—with China and Russia forming a separate, distinct clade from the other 19 countries in the sample.

## Discussion

### Transparency about aspirations and access

Across countries, measures to demonstrate the trustworthiness of the actors asking for and sharing genomic and health data should focus on transparency about the potential benefits of research, to whom such benefits will accrue and how this will happen. That is, it is not just about describing the general promise and potential of research—such as a benefit to future patients—but also providing an outline of how these benefits will be realised through research. While existing work has suggested the importance of a clear common goal in building trusting relationships with potential data donors, our findings are the first to emphasise the global importance of being clear in this message [16, 17].

It is also important to note, however, that transparency is not limited to outlining expected societal benefits (e.g. finding treatment to a disease) and how they are to be realised. A recurrent feature of research on public trust has been the low levels of trust associated with for-profit or private sector actors, particularly in relation to financial profits for shareholders [5–7]. Yet the benefits motivating the use of research or clinical access to large datasets are not only pecuniary. A further step towards demonstrating trustworthiness, and ‘respectful interactions’ [17] around data would be for potential data users to reflect on and acknowledge their multiple interests in and motivations for collecting and using health data, and to be transparent about these. Such transparency may enable data donors to more clearly understand what motivates the use of data, and thus make a more confident assessment of a data users’ motivation and the extent to which it reflects a desirable or common goal [10, 15].

A further role of transparency relates to the responsibility of those collecting and using genomic data to provide clear and accessible information on who is using data and for what purposes [28]. Our work suggests that such transparency is indeed an important feature of trustworthy data stewardship across the 22 countries studied. It also draws attention to the potential importance of familiarity, not just with genetics—as we have highlighted elsewhere—but with the individuals or organisations responsible for genomic data [22, 29].

Our findings suggest, however, that the value of openness does not necessarily extend to individuals’ ability to access their own DNA and health data. Such direct access as a reciprocation of individual contributions was comparatively less important to participants as a measure to increase trust. Thus, while access to data may be valued by some participants in light of personal interest and perceived personal utility, communicating the potential for such access is not necessarily likely to demonstrate the trustworthiness of actors using of genomic data.

### Data rights and regulation

While the measures discussed above concentrate on the goals and roles of genomic research and transparency about access to data, the high ranking of withdrawal and opt outs from data use highlights the importance and value of demonstrating adherence to research ethics frameworks, including informed consent. The right to withdraw, for example, is protected and reinforced throughout clinical research ethics guidance as well as data protection law (e.g. General Data Protection Regulation), but presents specific challenges in the case of long-term genomics research [1, 30]. Our findings suggest the value of efforts to reinforce and protect this right, even as data are de-identified and shared, making it clear to data donors that this right exists, but also to be open about its limits and the difficulties associated with tracking shared de-identified data.

The comparatively lesser importance attributed to sanctions for data misuse suggests that regulation and enforcement measures to prevent misuse and exploitation may be important to prevent a loss of trust but make a smaller contribution to demonstrating trustworthiness [14]. Respondents across all countries were less likely to value being able to communicate directly with the gatekeepers of genomic and health data collections, or to be able to access websites about data sharing or see profiles of researchers. The first is interesting, given the importance placed by policy-makers on identifying those responsible for data in regulatory interventions such as European Union’s General Data Protection Regulation (GDPR). The latter suggests that, a website might be of informational value to some, but that efforts to build trust may need more proactive engagement with data donors. This concords with work that suggests details about individual data users may have little value to data donors without knowledge of why that individual is trustworthy, returning discussion to the measures outlined above [31].

### Cross-country consistency and variation

While the overall picture provided by both the consolidated ranking and the individual country rankings grouped in Figs. 2 and 3 are consistent, our data show variation between countries in their views of measures to enhance trust. The option of communicating with gatekeepers, for example, was selected by a far higher proportion of respondents in Australia, Portugal and Russia than in Japan, Italy or India. Variation is also seen among countries that share, to an extent, legal and decision-making frameworks relevant to genomic data, such as member states of the European Union—although all ten European countries in the sample fall within the same broad clade of 17 countries in Fig. 4, each often has greatest similarity with countries outside the bloc. At a European level, these findings may contribute to the exploration of national variation in the implementation of regulation and legal safeguards derived from the GDPR [32].

Further patterns of consistency and divergence can be seen in the detail of the dendrogram. Some results appear intuitive while others are more unexpected. For example, the clade in which the UK and Australia cluster may be anticipated given their (partly) shared histories, and similar governance and social healthcare systems. The connection between France and Switzerland might also be expected, particularly among Francophone respondents. Other results are less expected —it is interesting that the USA is not as closely associated with some countries as might be anticipated given geographic proximity and/or shared histories, such as Canada or the UK. The clade that brings together India and Egypt with Japan, Russia and China is also suggestive of interesting directions for further investigation.

As a whole, the patterns of consistency and variation shown here provide nuance to discussions of trustworthiness and present challenges to those developing standards and governance models to facilitate the international sharing of genomic and health data. Most significantly, they suggest that while it is important to work to establish codes of conduct and demonstrate shared values and goals between researchers and data donors, conclusions from such work can only be tentatively extended across national settings. They further point to the need for detailed comparative work, including qualitative studies, to understand how and why trustworthiness can be demonstrated by the individuals and institutions using genomic and health data.

## Limitations

The limitations of the study and design have been published separately [24]. As an exploratory cross-sectional online survey, the study is limited in that it captures intended behaviour at a single time point. Three particular limitations are important to note. Firstly, our analysis is limited to those who would be willing to consider donating their DNA or health information for research. While this includes the majority of the sample, it cannot tell us which measures to increase trust may be more valued by those who definitely will not donate. Second, although the survey was translated and back-translated, nuances of language and culture may affect how participants interpret the options presented. Finally, measures deemed as important in this study, while important and likely necessary, are unlikely to be sufficient on their own to assure potential donors of DNA and health data of the trustworthiness of actors involved in collecting, using and sharing data.

## Conclusions

The analysis of responses across the 22 countries included in the Your DNA, Your Say survey suggests practical findings related to demonstrating the trustworthiness of genomic data initiatives, and directions for further research to explore global public perspectives in more detail. They show the importance of research ethics principles related to the right to withdraw, but suggest that legal and regulatory controls may be more important in preventing the loss of trust than in rebuilding it. While highlighting significant variation between countries, they emphasise the importance of transparency about data uses, but particularly about the goals of data collection, and the potential benefits for patients and society, and for data users themselves.

## Supplementary Information

**Additional file 1.** Additional tables and figures related to the Your DNA, Your Say sample and responses in pdf format.

## Data Availability

The full dataset is published at https://societyandethicsresearch.wellcomeconnectingscience.org/project/your-dna-your-say and available, without restriction, for anyone to access, download and analyse.
