# Supplementary material for: Demonstrating trustworthiness when collecting and sharing genomic data: public views across 22 countries
Source: Genome Med. 2021 May 25;13:92. doi: 10.1186/s13073-021-00903-0 (PMC8147072; doi:10.1186/s13073-021-00903-0)

**Additional File**

Table S1: Sociodemographic characteristics of country samples

| Variable | Categories | Total | | Argentina | | Australia | | Belgium | | Brazil | | Canada | | China | |
| --- | --- | --- | --- | --- | --- | --- | --- | --- | --- | --- | --- | --- | --- | --- | --- |
|  |  | N | % | N | % | N | % | N | % | N | % | N | % | N | % |
| Age category | 30 and under | 8719 | 24 | 233 | 25.4 | 200 | 16.5 | 90 | 16.5 | 412 | 30.5 | 634 | 21.4 | 907 | 30.2 |
|  | 31-40 | 8028 | 22.1 | 192 | 20.9 | 221 | 18.2 | 89 | 16.4 | 301 | 22.3 | 608 | 20.5 | 760 | 25.3 |
|  | 41-50 | 7044 | 19.4 | 159 | 17.3 | 228 | 18.8 | 105 | 19.3 | 268 | 19.9 | 528 | 17.8 | 507 | 16.9 |
|  | 51-60 | 6190 | 17.1 | 197 | 21.4 | 224 | 18.5 | 124 | 22.8 | 237 | 17.6 | 567 | 19.1 | 607 | 20.2 |
|  | Over 60 | 6287 | 17.3 | 138 | 15 | 339 | 28 | 136 | 25 | 131 | 9.7 | 629 | 21.2 | 227 | 7.5 |
| Gender | Female | 18526 | 51.1 | 485 | 52.8 | 656 | 54.1 | 290 | 53.3 | 723 | 53.6 | 1342 | 45.2 | 1434 | 47.7 |
|  | Male | 17742 | 48.9 | 434 | 47.2 | 556 | 45.9 | 254 | 46.7 | 626 | 46.4 | 1624 | 54.8 | 1574 | 52.3 |
| Has children | No | 13901 | 38.3 | 282 | 30.7 | 471 | 38.9 | 179 | 32.9 | 458 | 34 | 1316 | 44.4 | 833 | 27.7 |
|  | Yes | 21850 | 60.2 | 620 | 67.5 | 729 | 60.1 | 362 | 66.5 | 872 | 64.6 | 1607 | 54.2 | 2150 | 71.5 |
|  | Missing | 517 | 1.4 | 17 | 1.8 | 12 | 1 | 3 | 0.6 | 19 | 1.4 | 43 | 1.4 | 25 | 0.8 |
| Highest education level | Tertiary | 21488 | 59.2 | 589 | 64.1 | 650 | 53.6 | 245 | 45 | 874 | 64.8 | 1835 | 61.9 | 2017 | 67.1 |
|  | Secondary | 12302 | 33.9 | 280 | 30.5 | 433 | 35.7 | 264 | 48.5 | 366 | 27.1 | 931 | 31.4 | 876 | 29.1 |
|  | Primary | 1357 | 3.7 | 18 | 2 | 99 | 8.2 | 22 | 4 | 53 | 3.9 | 82 | 2.8 | 28 | 0.9 |
|  | Other | 1106 | 3 | 32 | 3.5 | 30 | 2.5 | 13 | 2.4 | 56 | 4.2 | 117 | 3.9 | 76 | 2.5 |
|  | Missing | 15 | 0 | 0 | 0 | 0 | 0 | 0 | 0 | 0 | 0 | 1 | 0 | 11 | 0.4 |
| Religiosity | Not a religious person | 19580 | 54 | 402 | 43.7 | 805 | 66.4 | 348 | 64 | 336 | 24.9 | 1742 | 58.7 | 2437 | 81 |
|  | A religious person | 16681 | 46 | 516 | 56.1 | 407 | 33.6 | 196 | 36 | 1013 | 75.1 | 1223 | 41.2 | 569 | 18.9 |
|  | Missing | 7 | 0 | 1 | 0.1 | 0 | 0 | 0 | 0 | 0 | 0 | 1 | 0 | 2 | 0.1 |

Table S1 (cont.)

| Variable | Categories | Egypt | | France | | Germany | | India | | Italy | | Japan | | Mexico | | Pakistan | |
| --- | --- | --- | --- | --- | --- | --- | --- | --- | --- | --- | --- | --- | --- | --- | --- | --- | --- |
|  |  | N | % | N | % | N | % | N | % | N | % | N | % | N | % | N | % |
| Age category | 30 and under | 658 | 46.1 | 133 | 16.8 | 250 | 21 | 149 | 30.9 | 218 | 17.7 | 620 | 13.1 | 409 | 30.4 | 494 | 53.4 |
|  | 31-40 | 386 | 27 | 129 | 16.3 | 171 | 14.3 | 126 | 26.1 | 215 | 17.5 | 711 | 15 | 337 | 25 | 277 | 29.9 |
|  | 41-50 | 265 | 18.6 | 139 | 17.6 | 225 | 18.9 | 109 | 22.6 | 301 | 24.5 | 955 | 20.1 | 293 | 21.8 | 115 | 12.4 |
|  | 51-60 | 94 | 6.6 | 139 | 17.6 | 237 | 19.9 | 54 | 11.2 | 237 | 19.3 | 976 | 20.6 | 181 | 13.4 | 27 | 2.9 |
|  | Over 60 | 24 | 1.7 | 250 | 31.6 | 310 | 26 | 44 | 9.1 | 258 | 21 | 1486 | 31.3 | 127 | 9.4 | 12 | 1.3 |
| Gender | Female | 709 | 49.7 | 421 | 53.3 | 635 | 53.2 | 192 | 39.8 | 627 | 51 | 2728 | 57.5 | 694 | 51.5 | 428 | 46.3 |
|  | Male | 718 | 50.3 | 369 | 46.7 | 558 | 46.8 | 290 | 60.2 | 602 | 49 | 2020 | 42.5 | 653 | 48.5 | 497 | 53.7 |
| Has children | No | 664 | 46.5 | 269 | 34.1 | 539 | 45.2 | 141 | 29.3 | 458 | 37.3 | 2012 | 42.4 | 503 | 37.3 | 469 | 50.7 |
|  | Yes | 678 | 47.5 | 514 | 65.1 | 646 | 54.1 | 338 | 70.1 | 761 | 61.9 | 2671 | 56.3 | 828 | 61.5 | 443 | 47.9 |
|  | Missing | 85 | 6 | 7 | 0.9 | 8 | 0.7 | 3 | 0.6 | 10 | 0.8 | 65 | 1.4 | 16 | 1.2 | 13 | 1.4 |
| Highest education level | Tertiary | 1176 | 82.4 | 408 | 51.6 | 353 | 29.6 | 152 | 31.5 | 351 | 28.6 | 2613 | 55 | 1159 | 86 | 834 | 90.2 |
|  | Secondary | 183 | 12.8 | 334 | 42.3 | 659 | 55.2 | 207 | 42.9 | 846 | 68.8 | 1938 | 40.8 | 103 | 7.6 | 72 | 7.8 |
|  | Primary | 24 | 1.7 | 28 | 3.5 | 134 | 11.2 | 76 | 15.8 | 18 | 1.5 | 21 | 0.4 | 4 | 0.3 | 9 | 1 |
|  | Other | 44 | 3.1 | 20 | 2.5 | 47 | 3.9 | 47 | 9.8 | 13 | 1.1 | 175 | 3.7 | 80 | 5.9 | 10 | 1.1 |
|  | Missing | 0 | 0 | 0 | 0 | 0 | 0 | 0 | 0 | 1 | 0.1 | 1 | 0 | 1 | 0.1 | 0 | 0 |
| Religiosity | Not a religious person | 280 | 19.6 | 545 | 69 | 775 | 65 | 42 | 8.7 | 443 | 36 | 3805 | 80.1 | 504 | 37.4 | 12 | 1.3 |
|  | A religious person | 1147 | 80.4 | 245 | 31 | 418 | 35 | 440 | 91.3 | 786 | 64 | 940 | 19.8 | 843 | 62.6 | 913 | 98.7 |
|  | Missing | 0 | 0 | 0 | 0 | 0 | 0 | 0 | 0 | 0 | 0 | 3 | 0.1 | 0 | 0 | 0 | 0 |

Table S1 (cont.)

| Variable | Categories | Poland | | Portugal | | Russia | | Spain | | Sweden | | Switzerland | | United Kingdom | | United States | |
| --- | --- | --- | --- | --- | --- | --- | --- | --- | --- | --- | --- | --- | --- | --- | --- | --- | --- |
|  |  | N | % | N | % | N | % | N | % | N | % | N | % | N | % | N | % |
| Age category | 30 and under | 742 | 25.6 | 550 | 24.7 | 338 | 31.4 | 177 | 13.9 | 168 | 20.5 | 59 | 17.7 | 912 | 26.8 | 366 | 17.5 |
|  | 31-40 | 628 | 21.6 | 665 | 29.9 | 425 | 39.5 | 249 | 19.6 | 120 | 14.6 | 38 | 11.4 | 691 | 20.3 | 689 | 32.9 |
|  | 41-50 | 599 | 20.6 | 623 | 28 | 225 | 20.9 | 256 | 20.1 | 153 | 18.6 | 66 | 19.8 | 619 | 18.2 | 306 | 14.6 |
|  | 51-60 | 542 | 18.7 | 281 | 12.6 | 73 | 6.8 | 203 | 16 | 133 | 16.2 | 73 | 21.9 | 588 | 17.3 | 396 | 18.9 |
|  | Over 60 | 393 | 13.5 | 105 | 4.7 | 14 | 1.3 | 387 | 30.4 | 247 | 30.1 | 97 | 29.1 | 597 | 17.5 | 336 | 16.1 |
| Gender | Female | 1486 | 51.2 | 1170 | 52.6 | 543 | 50.5 | 628 | 49.4 | 436 | 53.1 | 172 | 51.7 | 1713 | 50.3 | 1014 | 48.4 |
|  | Male | 1418 | 48.8 | 1054 | 47.4 | 532 | 49.5 | 644 | 50.6 | 385 | 46.9 | 161 | 48.3 | 1694 | 49.7 | 1079 | 51.6 |
| Has children | No | 1045 | 36 | 985 | 44.3 | 308 | 28.7 | 393 | 30.9 | 320 | 39 | 128 | 38.4 | 1480 | 43.4 | 648 | 31 |
|  | Yes | 1821 | 62.7 | 1199 | 53.9 | 744 | 69.2 | 870 | 68.4 | 490 | 59.7 | 201 | 60.4 | 1889 | 55.4 | 1417 | 67.7 |
|  | Missing | 38 | 1.3 | 40 | 1.8 | 23 | 2.1 | 9 | 0.7 | 11 | 1.3 | 4 | 1.2 | 38 | 1.1 | 28 | 1.3 |
| Highest education level | Tertiary | 1636 | 56.3 | 1335 | 60 | 967 | 90 | 817 | 64.2 | 359 | 43.7 | 107 | 32.1 | 1760 | 51.7 | 1251 | 59.8 |
|  | Secondary | 1157 | 39.8 | 752 | 33.8 | 84 | 7.8 | 379 | 29.8 | 348 | 42.4 | 161 | 48.3 | 1281 | 37.6 | 648 | 31 |
|  | Primary | 63 | 2.2 | 103 | 4.6 | 6 | 0.6 | 52 | 4.1 | 96 | 11.7 | 50 | 15 | 269 | 7.9 | 102 | 4.9 |
|  | Other | 48 | 1.7 | 34 | 1.5 | 18 | 1.7 | 24 | 1.9 | 18 | 2.2 | 15 | 4.5 | 97 | 2.8 | 92 | 4.4 |
|  | Missing | 0 | 0 | 0 | 0 | 0 | 0 | 0 | 0 | 0 | 0 | 0 | 0 | 0 | 0 | 0 | 0 |
| Religiosity | Not a religious person | 918 | 31.6 | 900 | 40.5 | 432 | 40.2 | 666 | 52.4 | 623 | 75.9 | 216 | 64.9 | 2524 | 74.1 | 825 | 39.4 |
|  | A religious person | 1986 | 68.4 | 1324 | 59.5 | 643 | 59.8 | 606 | 47.6 | 198 | 24.1 | 117 | 35.1 | 883 | 25.9 | 1268 | 60.6 |
|  | Missing | 0 | 0 | 0 | 0 | 0 | 0 | 0 | 0 | 0 | 0 | 0 | 0 | 0 | 0 | 0 | 0 |

Table S2: Number and percentage of participants who indicated they would not donate and therefore did not respond regarding measures to increase trust, by country.

| *Country* | *No* | *Yes* | *Percent No* |
| --- | --- | --- | --- |
| Argentina | 126 | 792 | 13.7 |
| Australia | 287 | 925 | 23.7 |
| Belgium | 113 | 431 | 20.8 |
| Brazil | 195 | 1151 | 14.5 |
| Canada | 628 | 2338 | 21.2 |
| China | 173 | 2835 | 5.8 |
| Egypt | 308 | 1117 | 21.6 |
| France | 146 | 644 | 18.5 |
| Germany | 226 | 967 | 18.9 |
| India | 20 | 462 | 4.1 |
| Italy | 164 | 1065 | 13.3 |
| Japan | 1579 | 3166 | 33.3 |
| Mexico | 120 | 1227 | 8.9 |
| Pakistan | 66 | 859 | 7.1 |
| Poland | 365 | 2539 | 12.6 |
| Portugal | 259 | 1965 | 11.6 |
| Russia | 234 | 841 | 21.8 |
| Spain | 158 | 1113 | 12.4 |
| Sweden | 157 | 664 | 19.1 |
| Switzerland | 54 | 279 | 16.2 |
| United Kingdom | 818 | 2589 | 24.0 |
| United States | 470 | 1623 | 22.5 |

Table S3: Pairwise correlations with 95% confidence intervals and p-values for strong (>=0.9) estimates; * indicates *p*-values below the Bonferroni-corrected threshold. The ‘Other’ category was not used in the correlation analysis as it is heterogeneous and cannot be meaningfully compared.

| Country 1 | Country 2 | Correlation | Lower 95% CI | Upper 95% CI | P-value |
| --- | --- | --- | --- | --- | --- |
| Mexico | Spain | 0.98 | 0.94 | 1.00 | 0.00015* |
| France | Poland | 0.98 | 0.94 | 1.00 | 0.00015* |
| Australia | Italy | 0.95 | 0.84 | 1.00 | 0.00022 |
| Australia | Canada | 0.93 | 0.81 | 1.00 | 0.00031 |
| Australia | UK | 0.93 | 0.81 | 1.00 | 0.00031 |
| Canada | France | 0.93 | 0.81 | 1.00 | 0.00031 |
| Canada | Poland | 0.93 | 0.80 | 1.00 | 0.00031 |
| France | Switzerland | 0.93 | 0.83 | 1.00 | 0.00031 |
| Poland | Switzerland | 0.93 | 0.81 | 1.00 | 0.00031 |
| Australia | Spain | 0.92 | 0.79 | 1.00 | 0.00042 |
| Argentina | Mexico | 0.91 | 0.78 | 1.00 | 0.00035 |
| Argentina | Sweden | 0.90 | 0.76 | 1.00 | 0.00046 |
| Australia | Mexico | 0.90 | 0.71 | 1.00 | 0.00046 |
| Belgium | Switzerland | 0.90 | 0.77 | 1.00 | 0.00046 |
| Mexico | Sweden | 0.90 | 0.76 | 1.00 | 0.00046 |

Figure S1: Proportion of respondents in each age group, by country


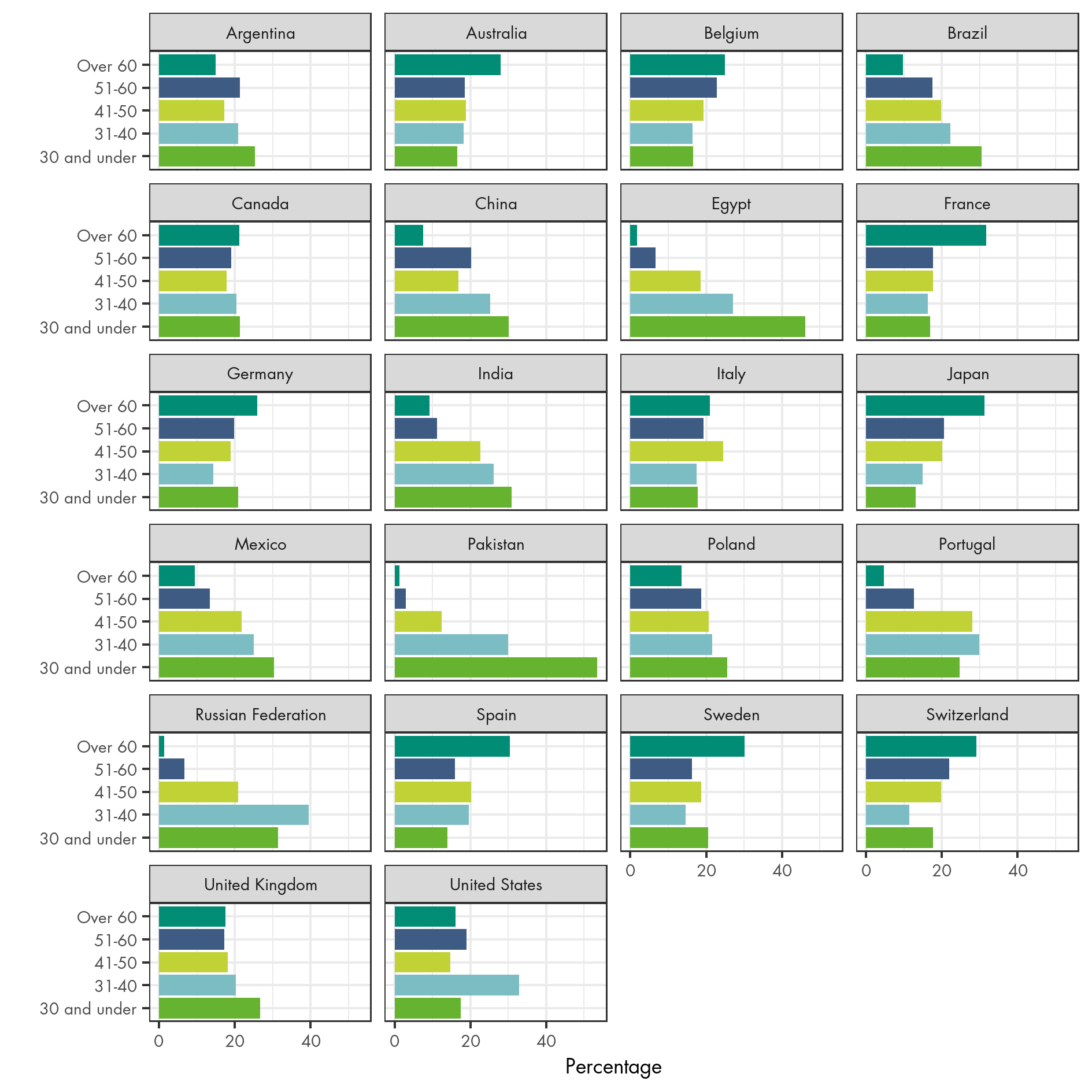


Figure S2: Proportion of respondents indicating they had children, by country


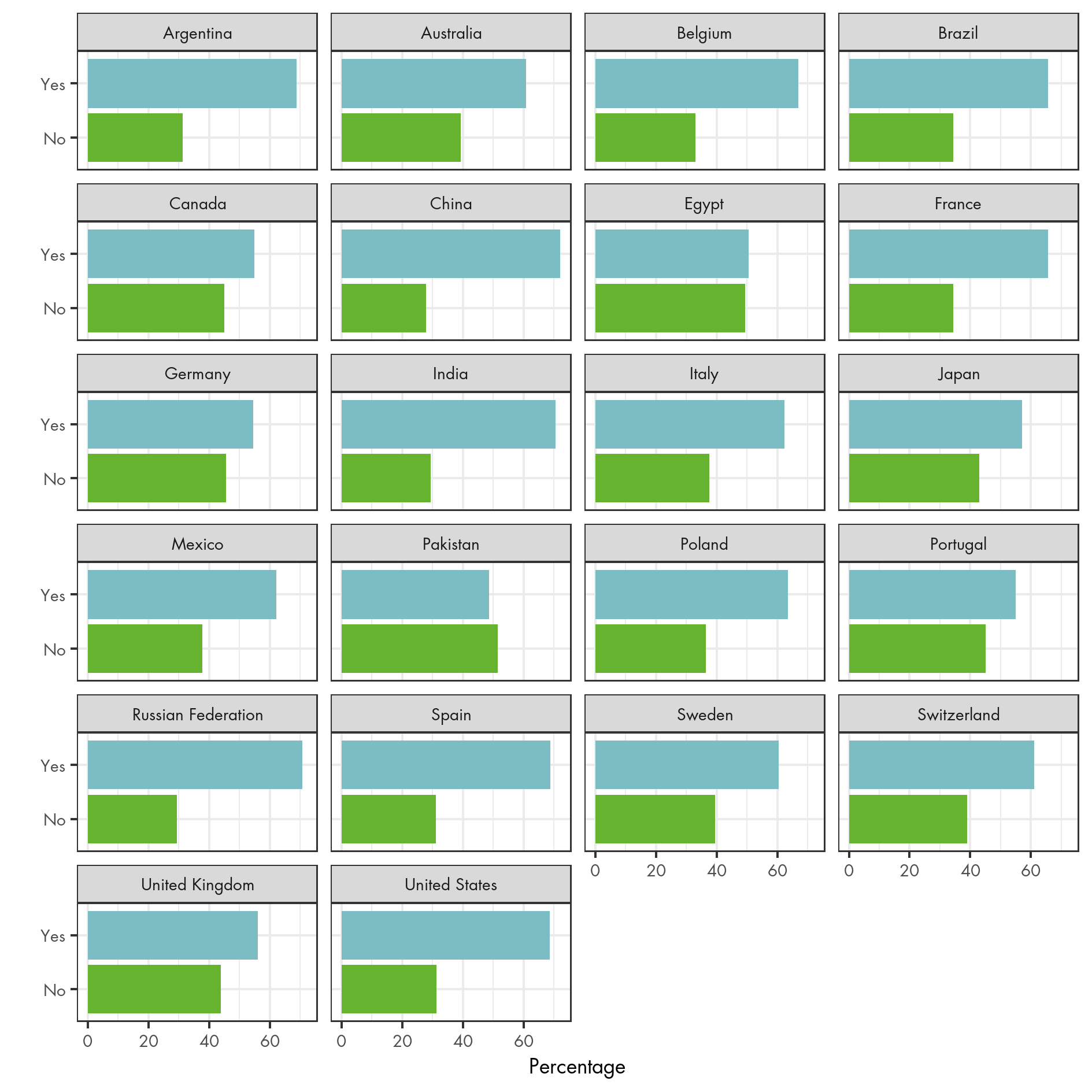


Figure S3: Proportion of respondents in each formal education category, by country


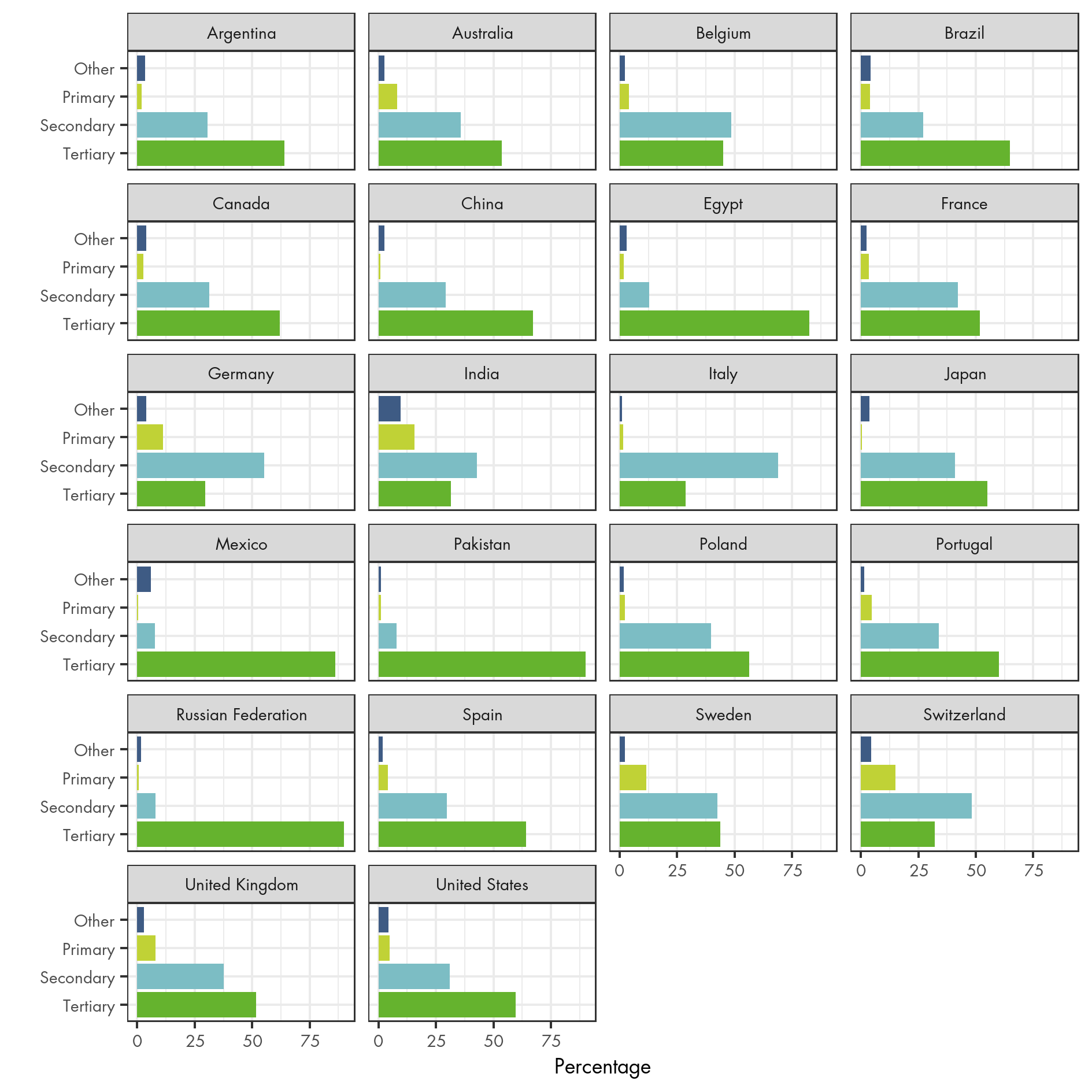


Figure S4: Gender of respondents, by country


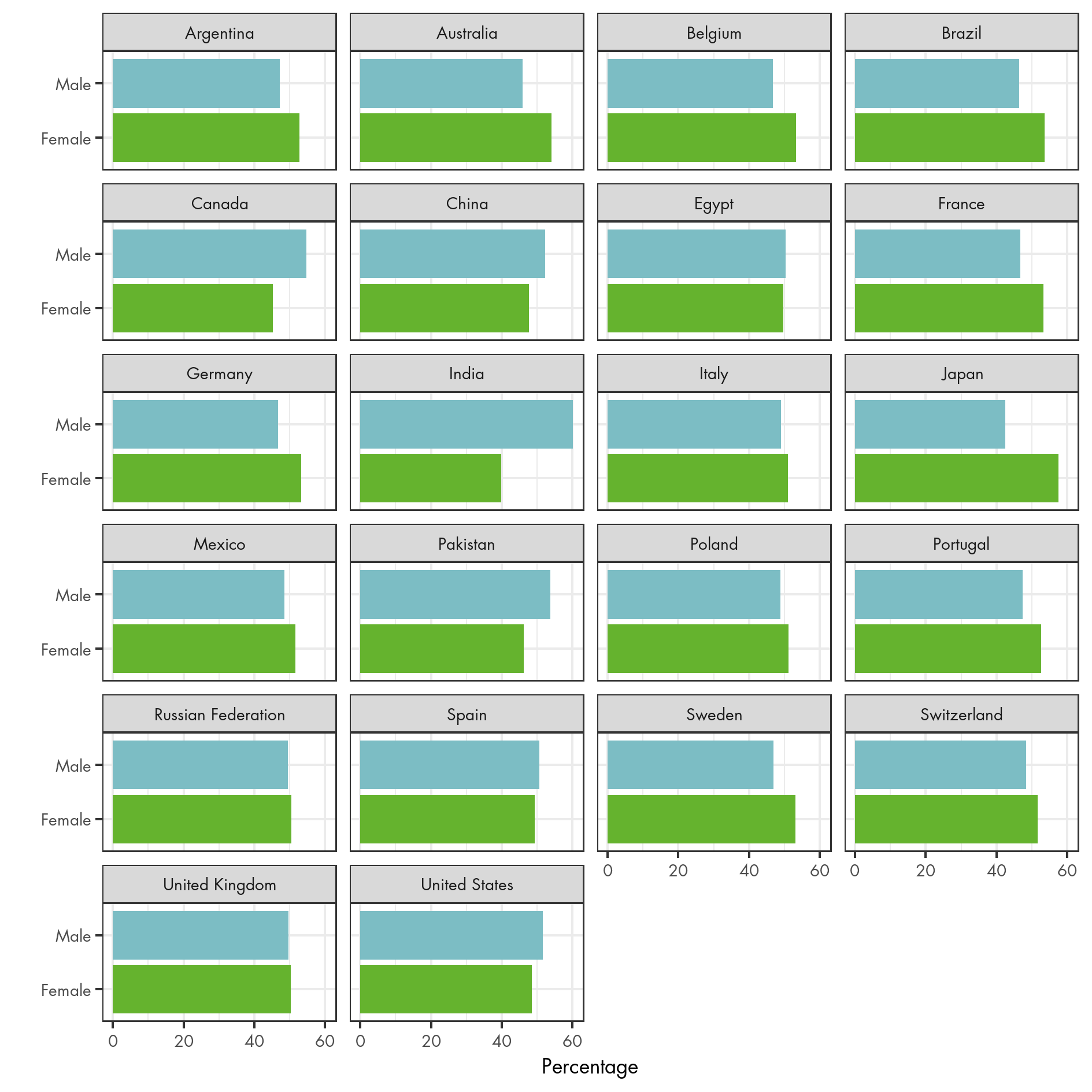


Figure S5: Proportion of respondents indicating that they were religious, by country


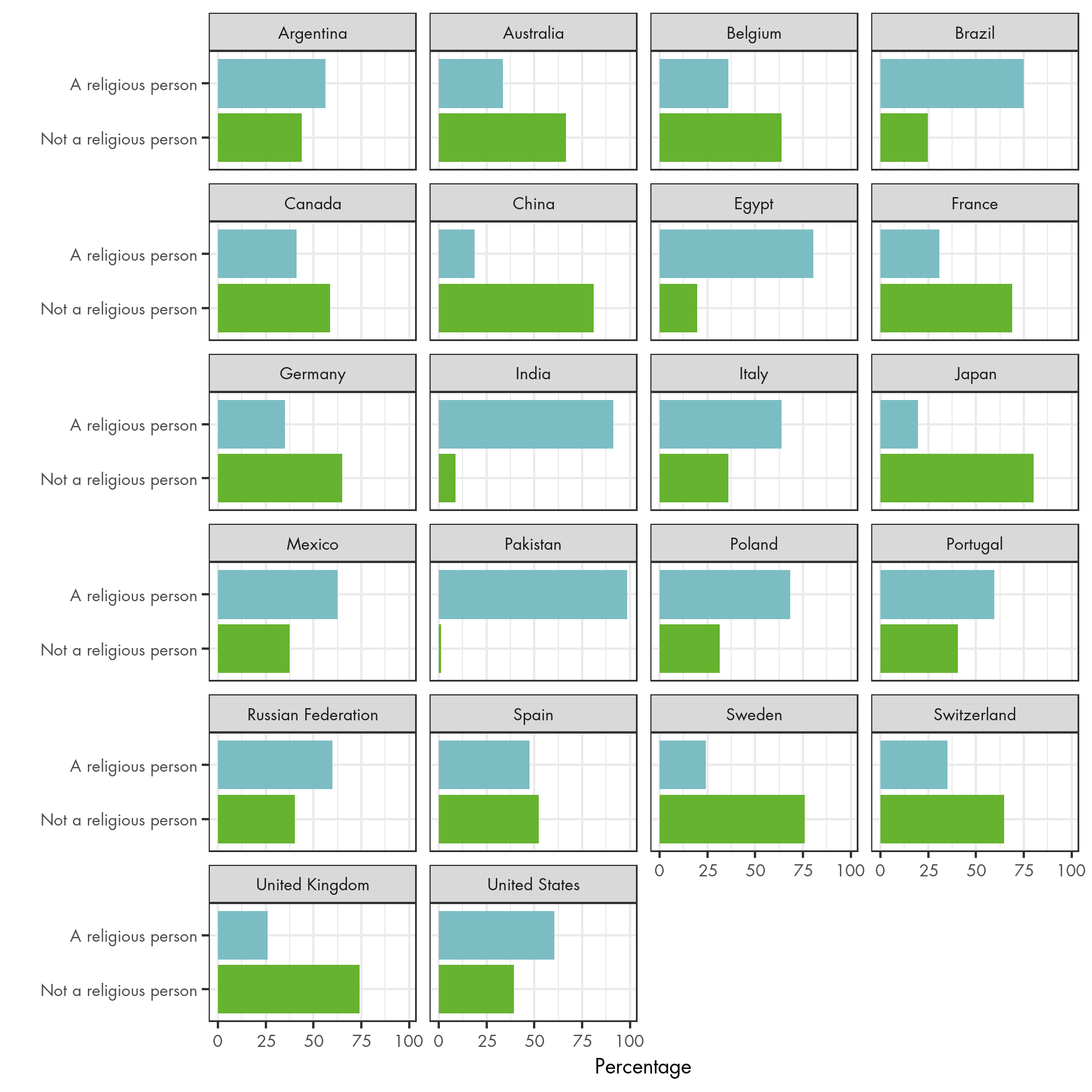

Supplement: Supplementary file 1 — Additional file 1. Additional tables and figures related to the Your DNA, Your Say sample and responses in pdf format. [file 13073_2021_903_MOESM1_ESM.docx]
